# Time-dependent effects of histone deacetylase inhibition in sepsis-associated acute kidney injury

**DOI:** 10.1186/s40635-020-0297-3

**Published:** 2020-02-07

**Authors:** Xiaoyan Wen, Shengnan Li, Alicia Frank, Xiukai Chen, David Emlet, Neil A. Hukriede, John A. Kellum

**Affiliations:** 1 Center for Critical Care Nephrology, Department of Critical Care Medicine, University of Pittsburgh School of Medicine, 3347 Forbes Ave, Suite 220, Pittsburgh, PA 15213 USA; 2 Department of Developmental Biology, University of Pittsburgh School of Medicine, Pittsburgh, PA USA

**Keywords:** Acute kidney injury, Renal tissue fibrosis, Sepsis, Histone deacetylase inhibitors

## Abstract

**Background:**

Sepsis, a dysregulated host response to infection with results in organ dysfunction, has been a major challenge to the development of effective therapeutics. Sepsis-associated acute kidney injury (S-AKI) results in a 3–5-fold increase in the risk of hospital mortality compared to sepsis alone. The development of therapies to reverse S-AKI could therefore significantly affect sepsis outcomes. However, the translation of therapies from preclinical studies into humans requires model systems that recapitulate clinical scenarios and the development of renal fibrosis indicative of the transition from acute to chronic kidney disease.

**Results:**

Here we characterized a murine model of S-AKI induced by abdominal sepsis developing into a chronic phenotype. We applied a small molecule histone deacetylase-8 inhibitor, UPHD186, and found that early treatment, beginning at 48 h post-sepsis, worsened renal outcome accompanied by decreasing mononuclear cell infiltration in the kidney, skewing cells into a pro-inflammatory phenotype, and increased pro-fibrotic gene expression, while delayed treatment, beginning at 96 h post-sepsis, after the acute inflammation in the kidney had subsided, resulted in improved survival and kidney histology presumably through promoting proliferation and inhibiting fibrosis.

**Conclusions:**

These findings not only present a clinically relevant S-AKI model, but also introduce a timing dimension into S-AKI therapeutic interventions that delayed treatment with UPHD186 may enhance renal histologic repair. Our results provide novel insights into successful repair of kidney injury and sepsis therapy.

## Background

Sepsis, a dysregulated host response to infection resulting in organ dysfunction [1], has proven to be a major challenge to the successful development of therapeutics. One obstacle has been the variation in mechanisms of organ injury and repair across organs [2]; another is the timing of organ injury which is often prior to patient presentation to medical care providers [3], which makes prophylactic treatment ineffective. One of the most common organ failures in sepsis is acute kidney injury (S-AKI) [4]. It results in a 3–5-fold increase in the risk of hospital mortality compared to sepsis without kidney injury [3]. Thus, the development of therapies to reverse S-AKI could have a major impact on sepsis outcomes.

Despite the tremendous effort and multiple candidates from experimental models to treat S-AKI, translation of therapies from preclinical studies into humans has been disappointing [5]. Two important reasons for this are the difficulty to deliver treatment designed to limit injury early enough and the lack of efficacy in facilitating kidney repair. These deficiencies are compounded by the fact that patients often present with S-AKI after the injury has already occurred [3], and the development of maladaptive repair is often insidious. However, when renal recovery does occur, survival is dramatically enhanced [6–10].

No effective treatment has been developed for S-AKI or for sepsis in general. Interventions have mainly focused on reducing organ damage, whereas facilitating recovery has received less attention. Histone deacetylase inhibitors (HDIs) modulate a variety of biological processes through modifying histone and non-histone targets; they regulate inflammation reactions [11–13] and therefore may have roles in affecting outcomes from sepsis. We recently discovered a novel class of HDIs, phenylthiobutanoic acids (PTBAs), that, through modulating HDAC8, regulate cell cycle progression after injury [14]. Our previous work has shown that a PTBA prodrug, UPHD186, promotes regeneration after AKI in zebrafish [15] and decreases fibrosis in a murine AKI model even when administered days after the initial insult [16–18]. However, the role of UPHD186 in tissue repair after S-AKI is unknown.

Here we characterized a murine model of S-AKI induced by abdominal sepsis developing into a chronic kidney disease-like phenotype and examine treatment effects of UPHD186 on renal outcomes. Specifically, we applied UPHD186 either before (48 h from CLP) or after (96 h from CLP) the resolution of inflammation as shown by expression of circulating IL-6 (interleukin-6) and renal neutrophil gelatinase-associated lipocalin (NGAL). We found that early treatment with UPHD186 further worsened kidney damage whereas delayed treatment improved survival and renal histology and decreased development of fibrosis. Our results are highly relevant to S-AKI in humans. We recently reported that, in patients admitted with a diagnosis of septic shock, 50% had clinical evidence and an additional 20% had biomarker-only evidence of AKI when they presented to medical attention [3]. When comparing these patients to patients without AKI, we found that in-hospital survival was highly associated with S-AKI. Furthermore, long-term follow-up (1–3 years) in these same patients and in other similar populations [10] has also shown that, regardless of the initial AKI staging, the extent of kidney recovery is a significant determinant of overall survival, emphasizing the importance of promoting recovery in S-AKI treatment.

## Results

### S-AKI develops into a chronic kidney disease (CKD)-like phenotype

**Fig. 1:**
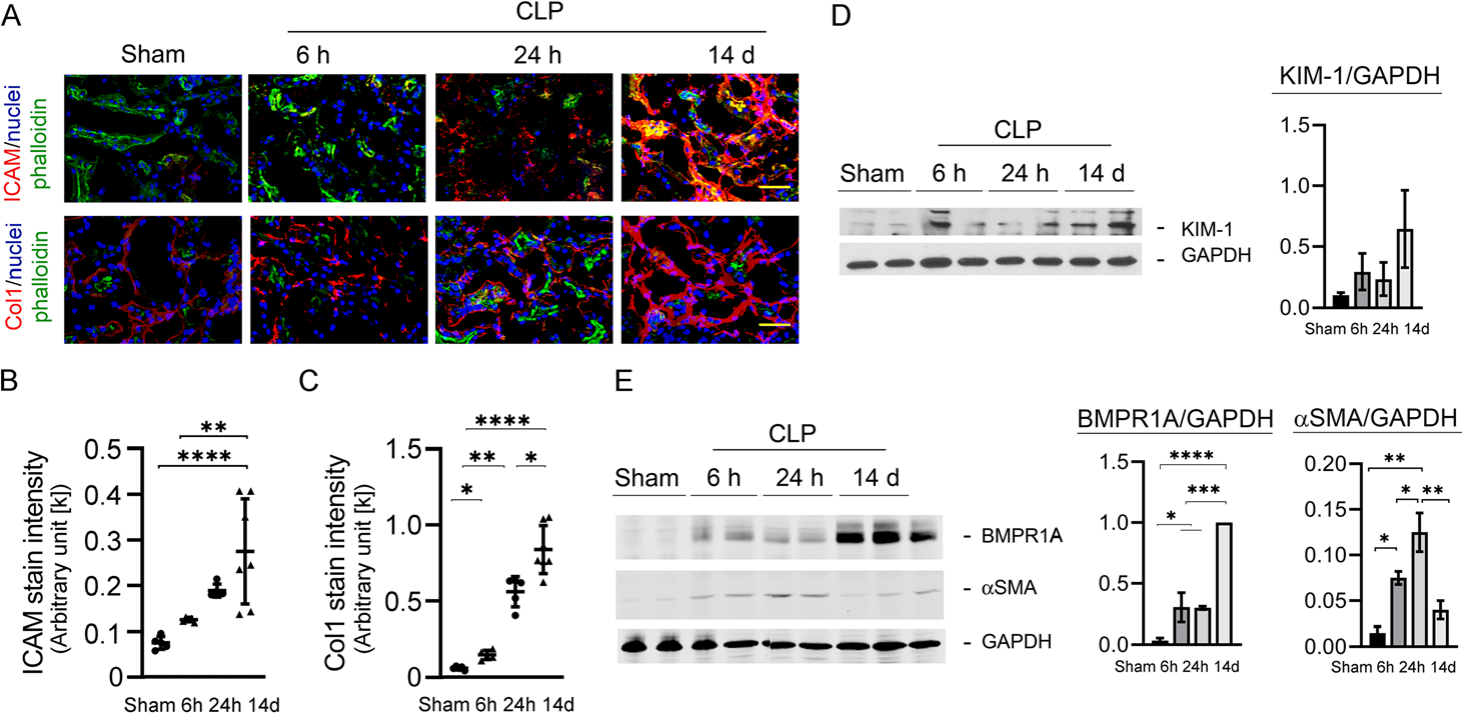
SA-AKI non-recovery and treatment timing. A pro-fibrotic S-AKI outcome at 2 weeks post the initial insult. Sepsis was induced by cecal ligation and puncture surgery (CLP) and the renal expressions of injury markers were shown. **a**–**c** Representative images of renal intercellular adhesion molecule (ICAM) and collagen type I (Col1) staining and the expression intensities for group animals (mean ± SD, *N* = 6~7). Scale bar = 30 μm. **d**–**e** Blots of kidney injury marker expressions and the corresponding intensities of the blots. Tukey’s test was used to determine the statistical significance. *P < 0.05, **P < 0.01, ***P < 0.001, ****P < 0.0001. KIM-1 results are for illustrative purposes. KIM-1, kidney injury molecule-1; BMPR1A, bone morphogenetic protein receptor type 1A; αSMA, alpha-smooth muscle actin; GAPDH, glyceraldehyde 3-phosphate-dehydrogenase

Aged Balb/c mice (20–24 weeks old, both male and female) were subjected to cecal ligation and puncture (CLP) surgery and followed for 14 days for survival and signs of maladaptive repair in the kidneys. Renal expression of intercellular adhesion molecule (ICAM-1) and collagen type I (Col1) were examined at 6 h, 24 h, and 14 days by immune staining. Representative images show increased stain intensities for ICAM-1 and Col1 at day 14 comparing to earlier time points (Fig. 1a). The quantified intensities for group animals are significantly increased on day 14 (ICAM-1, 14 days 274.6 ± 115.2 vs. sham 74.9 ± 13.9, *P <* 0.0001; Col1, 14 days 836.5 ± 158.4 vs. sham 59.8 ± 11.5, *P <* 0.0001; Fig. 1b, c), suggesting activated renal inflammation and collagen synthesis. Consistently, kidney tissue homogenate western blots and the corresponding kidney injury molecule-1 (KIM-1) and bone morphogenetic protein receptor type 1A (BMPR1A) also show increased protein expression on day 14 relative (KIM-1/GAPDH, 14 days 0.65 ± 0.32 vs. 24 h 0.24 ± 0.13, *P =* 0.27; BMPR1/GAPDH, 14 days 1.0 ± 0.01 vs. 24 h 0.3 ± 0.01, *P <* 0.001), whereas alpha-smooth muscle actin (αSMA) transiently peaked around 6~24 h and subsided thereafter (αSMA/GAPDH, 24 h 0.13 ± 0.02 vs. 6 h 0.08 ± 0.01, *P <* 0.05; 14 days 0.04 ± 0.01 vs. 24 h 0.13 ± 0.02, *P <* 0.01) (Fig. 1d, e). These findings are indicative of maladaptive repair [19], a CKD-like phenotype post-AKI.

### Determination of treatment timing based on resolution of inflammation and kidney dysfunction

**Fig. 2:**
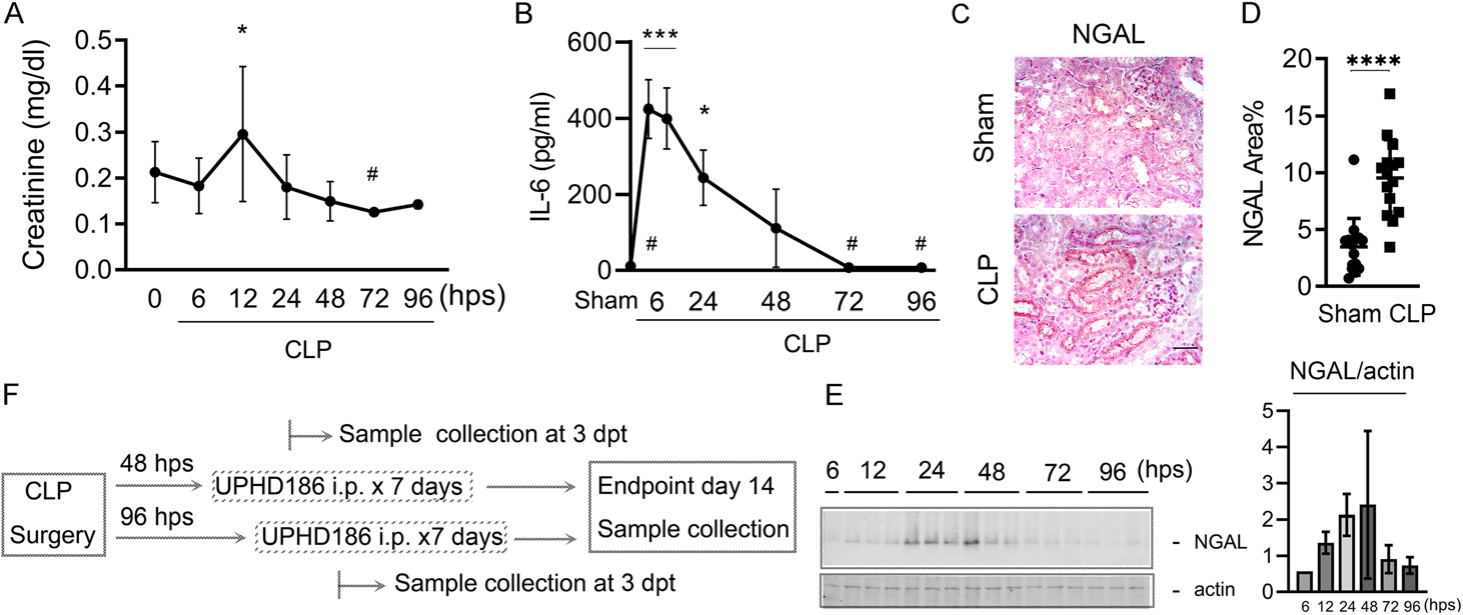
Treatment window. Determination of treatment timing based on resolution of renal dysfunction and systemic inflammation. Time serial samples were obtained at 6~96 h after the initial insult. **a**–**b** Serial charts of creatinine and the circulating interleukin 6 (IL6) are shown (mean ± SD, *N* = 3~5) *P < 0.05, ***P < 0.001 compared to “#.” **c**–**d** Representative images of immune stain for NGAL in the kidneys collected at 48 h post-CLP (scale bar = 20 μm) and the expression intensities for group each group of animals (mean ± SD, *N* = 15). **e** Blots of renal time serial NGAL and the corresponding intensities of the blots. **f** Schematic diagram of treatment timing and sample collection plan. Tukey’s test was used to determine the statistical significance among multiple groups and one-tailed Welch’s *t* test was used for two group comparisons. NGAL, neutrophil gelatinase-associated lipocalin; hps, hours post-CLP surgery; i.p., intraperitoneal injection; dpt, days post-UPHD186 treatment

To delineate the time course of S-AKI pathology, serial blood and renal tissue samples were collected over 6–96 h following initial injury and subjected to creatinine and cytokine assays. Although serum creatinine only briefly increased (72 h 0.3 ± 0.001 vs. 12 h 0.1 ± 0.2 mg/dl, *P* < 0.05; Fig. 2a), our results show robust inflammatory reactions. Specifically, circulating pro-inflammatory cytokines interleukin (IL)-6 peaked at ~ 6–12 h post-CLP and then returned to baseline by 72 h (72 h 7.8 ± 1.8 vs. 6 h 424.3 ± 76.5 pg/mL, *P* < 0.001; Fig. 2b); renal expression of NGAL was increased in CLP (48 h) animals compared to sham and stain intensity analysis across groups shows highly significant differences (CLP vs. sham 9.5 ± 3.4 vs. 3.5 ± 2.5, *P* < 0.0001; Fig. 2c, d). NGAL peaked at ~ 24–48 h (NGAL/actin, 96 h 0.7 ± 0.2 vs. 48 h 2.4 ± 2.0, *P =* 0.36; Fig. 2e). All inflammatory indicators return to normal by 72 h. Based on these results, we set time to initiation of treatment at either 48 h (early treatment) or 96 h (delayed treatment) post-CLP surgery, before or after the resolution of IL-6 and NGAL. Treatment effects were checked at 3 days post-treatment and at the CLP-day-14 endpoint (Fig. 2e).

### Effects of UPHD186 on kidney monocytes and their phenotype

**Fig. 3:**
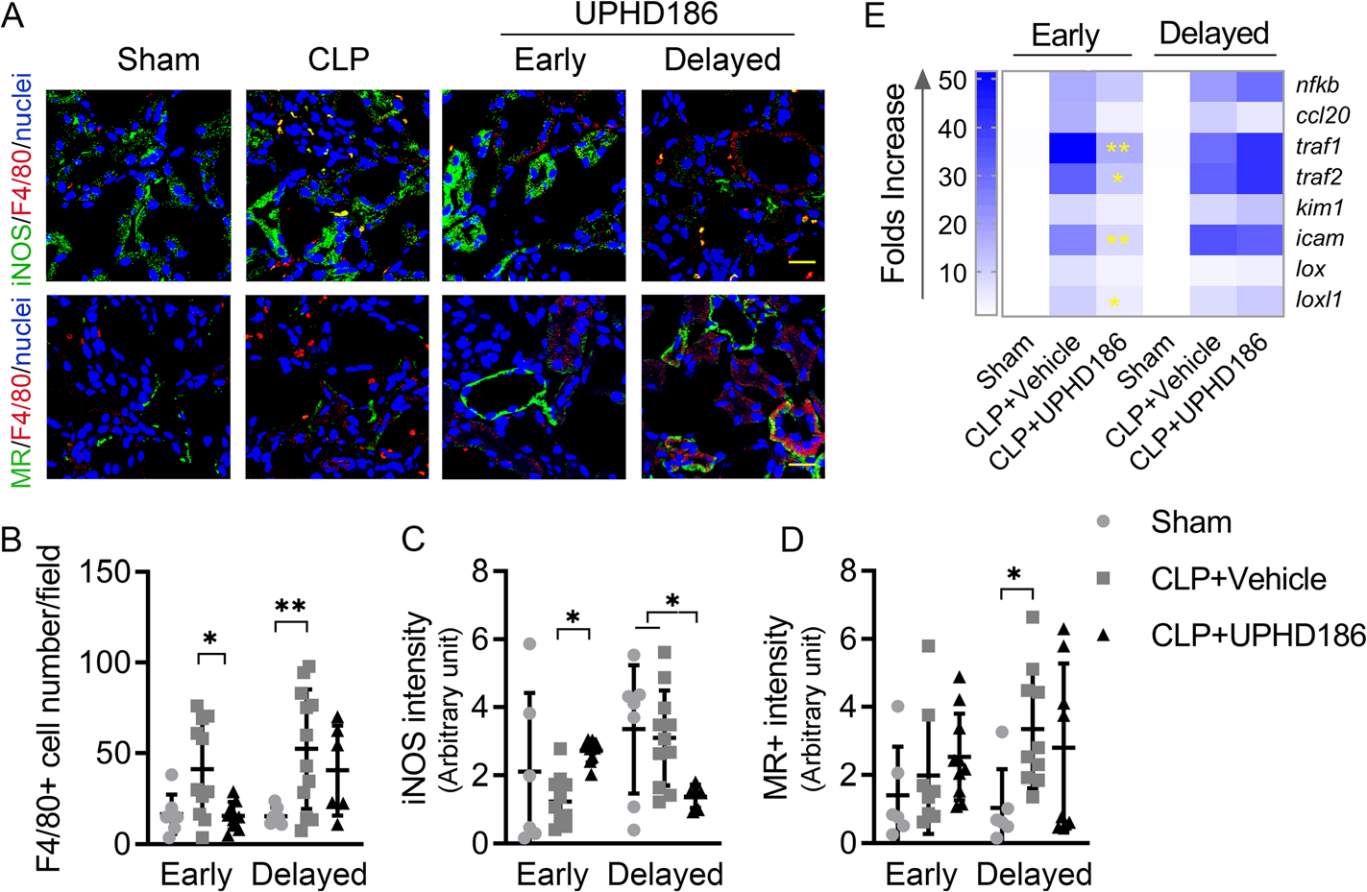
Short-term effect. Treatment effects measured at treatment day 3. **a** Representative images of immune staining of markers of mononuclear cells and their phenotype. Scale bar = 20 μm. **b**–**d** Dot plots of F4/80^+^ MNC cell numbers per field, the stain intensities of iNOS^+^ and MR^+^ colocalized with F4/80^+^ for group animals (mean ± SD, *N* = 7~12). Tukey’s test was used to determine statistical significance. **e** Heat map of renal mRNA expressions of inflammation and matrix remodeling mediator markers (*N* = 6~8). *P < 0.05, **P < 0.01 compared to the CLP + vehicle group. iNOS, inducible nitric oxide synthases; F4/80, also known as EMR1, EGF-like module-containing mucin-like hormone receptor-like 1; MR, mannose receptor; *nfkb*, nuclear factor kappa B; *ccl10*, C-C Motif Chemokine Ligand 20; *icam*, intercellular adhesion molecule; *traf*, tumor necrosis factor receptor-associated factor; *kim1*, also known as *HAVCR1*, hepatitis A virus cellular receptor 1; *lox*, lysyl oxidase; *loxl1*, lysyl oxidase homolog 1

Kidney samples collected at day 3 post-treatment were examined for presence of numbers of monocytes in the kidney and their phenotypes (monocytes, F4/80^+^; M1, F4/80^+^iNOS^+^; M2, F4/80^+^MR^+^). Our results show that compared to sham, septic animals had increased numbers of F4/80^+^cells in the kidneys (early treatment animals: sham 16.6 ± 10.8 vs. CLP 41.2 ± 26.1 cells/field, *P =* 0.07; delayed treatment animals: sham 15.5 ± 4.8 vs. CLP 52.4 ± 32.9 cells/field, *P <* 0.01). Early treatment with UPHD186 significantly inhibited this increase (15.7 ± 7.7 cells/field, comparing to the corresponding CLP, *P <* 0.05), whereas delayed treatment had no significant effect (40.6 ± 24.7 cells/field, *P =* 0.55; Fig. 3a, b). Interestingly, early and delayed treatment had opposite effects on iNOS expression (a marker of M1 phenotype) compared to CLP vehicle-treated animals with the early treatment increasing the expression intensities (2.7 ± 0.3 vs. CLP 1.2 ± 0.7, *P* < 0.05) and delayed treatment decreasing the expression intensities (1.4 ± 0.3 vs. CLP 3.1 ± 1.4, *P* < 0.05) (Fig. 3a, c). For MR, a marker of M2 phenotype, with its postponed increase seeing in the delayed CLP group (CLP vs. sham 3.3 ± 1.7 vs. 1.0 ± 1.1, *P <* 0.05), no treatment effects were observed in both groups (Fig. 3a, d). These results indicated that early treatment may prohibit monocyte infiltration into the kidneys and tip the balance toward M1, whereas delayed treatment does not affect the renal monocyte infiltration and tended to inhibit M1 phenotype transition.

### Effects of UPHD186 on markers of inflammatory gene expression, kidney injury, and matrix remodeling

Kidney samples were examined at mRNA levels for genes controlling inflammation (*nfκb*, *ccl20*, *traf1*, *traf2*, *icam*), kidney injury (*kim1*), and matrix remodeling markers (*lox*, *loxl1*). Septic animals had higher levels of gene expression compared to sham controls (Additional file 1: Figure S1). Early treatment significantly inhibited increases in 4 of 8 gene products (fold increase: *traf1* early treatment 17.1 ± 3.6 vs. CLP 51.5 ± 9.1, *P <* 0.01; *traf2*, 12.2 ± 3.0 vs. 32.5 ± 6.9, *P <* 0.05; *icam1*, 9.1 ± 2.3 vs. 25.3 ± 4.1, *P <* 0.01; *loxl1*, 5.0 ± 2.3 vs. 10.5 ± 5.7, *P <* 0.05), whereas delayed treatment had no effect on any marker expressions compared to CLP controls (Fig. 3e).

### Effects on sepsis survival, circulating creatinine, and cytokines

**Fig. 4:**
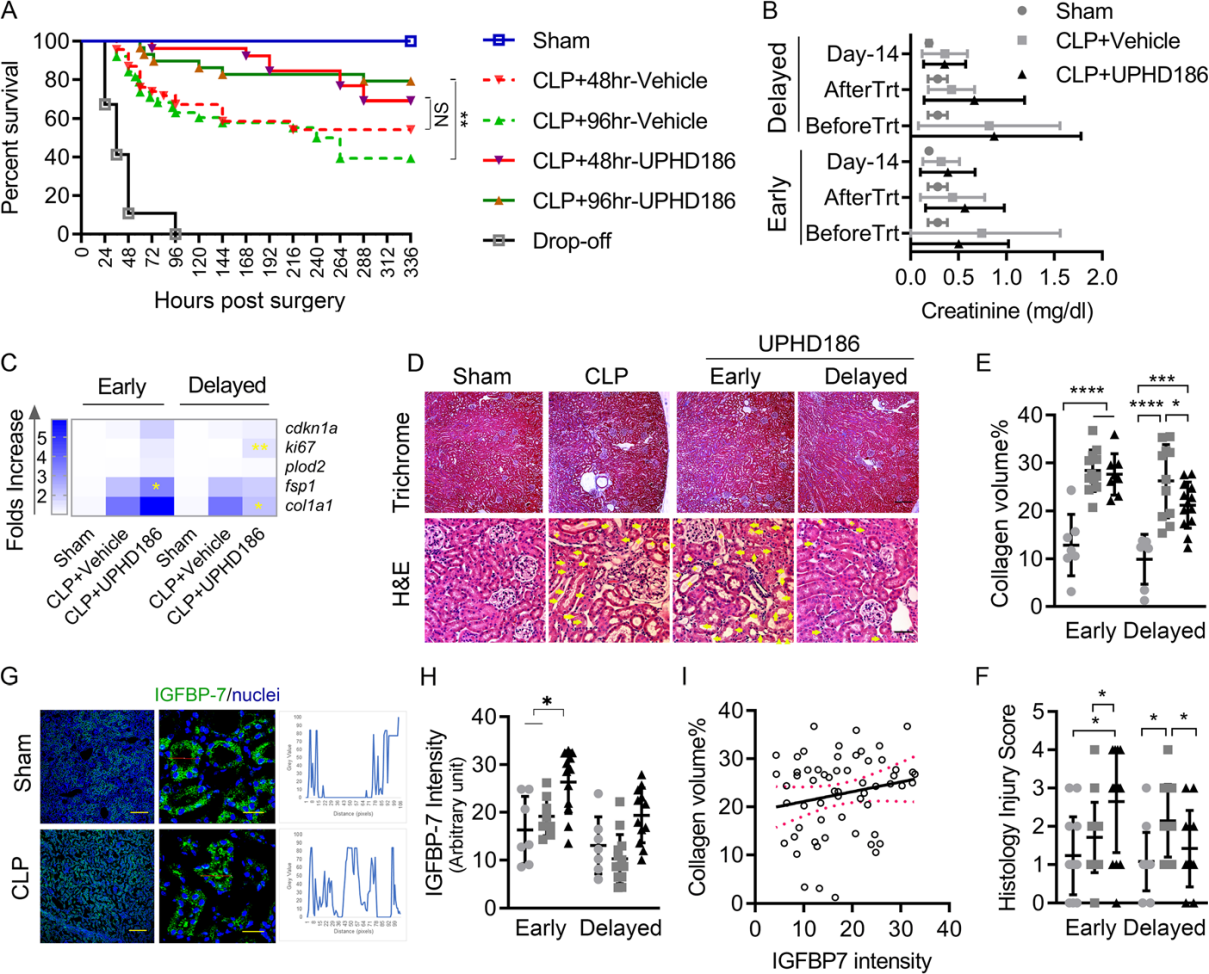
Two-week outcome. Treatment effects at 14-day endpoint comparing early versus delayed treatment effects. **a** Kaplan-Meier survival curves. Animals dying prior to treatment (at 48 or 96 h) are shown as “drop-off.” More animals died prior to the 96-h time point and thus all comparisons are between UPHD186 and vehicle and not across treatment regimens. **b** Circulating creatinine measured at 15 min before initiation of treatment (before Trt), 1 day after the 7-day treatment session (after Trt), and the endpoint. **c** A heat map of renal mRNA expressions of the fibrosis proliferation markers (*N* = 9~12). *P < 0.05, **P < 0.01 compared with the CLP + vehicle group. **d** Representative images of renal histochemistry staining. Scale bars = 200 μm for trichrome and 20 μm for H&E stain. **e**–**f** Dot plots of fibrosis positive stain area% and renal tubule injury scores for group animals (mean ± SD, *N* = 7~13). **g** Representative immune stain images of IGFBP7 in the kidney. Scale bars = 200 μm for low-power field and 30 μm for high-power field. The plots in blue at the right side show the intensity of IGFBP-7 stain across the red line pixel scan denoted in the high-power field image. **h** Dot plots of IGFBP-7 stain areas% for group animals (mean ± SD, *N* = 7~13). **i** Linear regression plot of renal IGFBP-7 and collagen volume%. Equation *Y* = 0.2 × *X* + 19.1, *R*^2^ = 0.04, *P* = 0.15. To calculate statistical significance, differences of survival rates were analyzed using log-rank Mantel-Cox test; Tukey’s test was used for multiple-group differences and one-tailed Welch’s *t* test for two group comparisons. NS, no statistical difference; *P < 0.05, **P < 0.01, ***P < 0.001. Sham groups were pooled when comparable. IGFBP7, insulin-like growth factor-binding protein 7; *cdkn1a*, cyclin-dependent kinase inhibitor 1a; *ki67*, Ki-67; *plod2*, procollagen-lysine, 2-oxoglutarate 5-dioxygenase 2; *fsp1*, fibroblast-specific protein 1; *col1a1*, collagen type I alpha 1 chain

As expected, sepsis animals had lower survival rates by day 14 compared to sham CLP + vehicle starting at 48 h (early) 54%, CLP + vehicle starting at 96 h (delayed) 39.0%, and sham 100%, *P <* 0.0001 comparing CLPs to sham group. Early treatment did not show beneficial effects (69% vs. 54%, *P* = 0.32), whereas delayed treatment resulted in significantly improved in sepsis survival (79% vs. 39%, *P* < 0.01) (Fig. 4a). However, creatinine levels across the treatment period were not different between groups (Fig. 4b) nor were circulating IL6 concentrations affected by treatment (Additional file 1: Figure S2).

### Effects on markers of cell cycle regulation, proliferation, and fibrosis

Kidney samples on day 14 (following CLP) were examined for mRNA levels for treatment effects on cell cycle regulation (*cdkn1a*), proliferation (*ki67*), and pro-fibrosis genes (*plod2*, *fsp1*, *col1a1*). Septic animals had higher levels of *fsp1* and *col1a1* expressions compared to sham controls (Additional file 1: Figure S3). Early treatment with UPHD186 further increased *fsp1* (fold increase, 3.3 ± 1.3 vs. corresponding CLP + vehicle controls 2.2 ± 1.1, *P <* 0.05), whereas delayed treatment promoted *ki67* and inhibited *col1a1* expression (fold increase, *ki67* 1.6 ± 0.7 vs. corresponding controls 1.0 ± 0.5, *P <* 0.01; *col1a1*, 2.2 ± 0.9 vs. 3.7 ± 2.4, *P <* 0.05) (Fig. 4c). Thus, pro-fibrotic gene expression was increased in the recovery phase and early treatment further aggravated these changes, whereas delayed treatment promoted proliferation and attenuated fibrosis.

### Delayed treatment with UPHD186 decreased tissue collagen volume and reduced fibrosis

At day 14 following CLP, renal Masson’s trichrome staining showed patchy collagen fibers in renal interstitium; hematoxylin and eosin staining displayed signs of tissue destruction (marked by yellow arrows), including disrupted renal cell arrangement and epithelial cell nuclei-plasma dissociation, cast formation, and tubule dilation (marked by yellow asterisk, Fig. 4d). Collagen volume fraction and renal histology injury score were higher in septic animals compared to sham controls: collagen volume fraction%—early treated group, CLP 28.43 ± 4.3 vs. sham 12.9 ± 6.4, *P <* 0.0001; delay treated group, 26.2 ± 7.6 vs. 9.9 ± 5.2, *P* < 0.0001; histology injury score—early treated group, CLP 1.7 ± 0.9 vs. sham 1.2 ± 1.0, *P* = 0.4; delay treated group, 2.1 ± 0.9 vs. 1.0 ± 0.8, *P* < 0.05. Whereas early treatment had no effects on fibrosis (collagen volume, 27.6 ± 4.3 vs. 28.4 ± 4.3, *P* > 0.05), the treatment aggravated histology injury (2.6 ± 1.3 vs. 1.7 ± 0.9, *P <* 0.05). Delayed treatment tended to decreased collagen volume (collagen volume fraction% 21.2 ± 4.8 vs. vehicle 26.2 ± 7.6, *P <* 0.05) and reduced histologic injury (histology injury score 1.4 ± 1.0 vs. vehicle 2.1 ± 1.0, *P <* 0.05) (Fig. 4e, f). Taken together, these results are consistent with mRNA measurement, confirmed the effects of delayed treatment on reducing tissue injury and inhibiting fibrosis.

Renal expression of the stress marker insulin-like growth factor-binding protein 7 (IGFBP7) was measured at 14 days post-CLP. In septic animals, IGFBP7 protein expression was anatomically displaced with higher-level staining appearing in the tubule lumen compared to sham controls (Fig. 4g). Although neither treatment regimens changed the secretory pattern of IGFBP7 (data not shown), early treatment significantly increased the overall IGFBP7 renal expression (early treated 674.1 ± 190.5 vs. CLP 396.9 ± 109.0, *P* < 0.05), whereas delayed treatment had no such effect (Fig. 4h). Linear regression analysis of correlated IGFBP7 with collagen volume% indicates a positive association between these two indicators but not achieving statistical significance *P* = 0.15 (Fig. 4i).

## Discussion

Once controversial [8], it is now generally accepted that AKI leads to CKD [20]. Often, this relationship is obvious—CKD results when renal function remains low following an AKI episode. However, more complicated patterns of recovery following AKI have been reported [7] and concerns have been raised that renal functional recovery may belie ongoing insidious injury or progression [6]. Thus, AKI may develop into CKD, even in the cases where there is an appearance of renal recovery measured by conventional renal indicators. Models of ischemia-reperfusion [16] and nephrotoxic AKI [21] that develop a CKD-like fibrosis phenotype have been reported. However, sepsis, the most common form of AKI in critically ill humans, has not, until now, enjoyed a viable preclinical model that recapitulates the maladaptive repair survivor phenotype.

Here we report the characteristics of a model of S-AKI in mice that develops into a pro-fibrotic, CKD-like phenotype (Fig. 1). Our model includes antibiotics and fluid resuscitation and is carefully titrated to be “just severe enough” to cause significant AKI and yet allow for sufficient survival. In this model, serum creatinine briefly increased, and circulating cytokine levels were transiently increased with both parameters returning to normal within 72 h (Fig. 2a, b, and e). Yet underlying cellular pathophysiological processes, increased fibrosis gene expression, interstitial collagen deposition, and injury marker expression in the kidney were documented and were found associated with increased mortality (Figs. 3e and 4a, c–f).

To demonstrate the utility of this model, a small molecule, PTBA, was administered as the prodrug UPHD186. Our previous work with this compound has shown efficacies in models of unilateral ureteric obstruction [16], nephrotoxic AKI [17], and in ischemia-reperfusion injury [18]. The drug modulates a wide range of cellular functions [11, 22] and has been reported to promote renal histology by expanding the renal progenitor cell population in zebrafish AKI models and reducing renal interstitial fibrosis in murine fibrotic kidney injury models when administered days after the initial insults [15–18]. Given these effects, we hypothesized a time-dependent treatment efficacy in S-AKI and sought to test this hypothesis when given either before or after inflammation had subsided. Treatment effects were examined at two time points during and after the resolution of kidney inflammation, which included monocyte biology, inflammatory marker expressions on the third day of therapy, and cellular events and fibrosis at day 14. Because the two drug regimens (starting from 48 h to 96 h post the CLP) were applied to different animals—those in the late group had to survive 48 h longer before treatment, comparisons of treatment efficacy can only be performed with the corresponding vehicle group initiated at the same time point, but not across the two treatment strategies.

Our results show a positive treatment impact on sepsis survival with delayed administration of UPHD186, together with increased indicators of tissue proliferation and reduced fibrosis. Interestingly, serum creatinine and cytokines were insensitive to these pathological features—consistent with clinical observations [6, 7]. Maladaptive repair and fibrosis are important mechanisms leading to CKD, apparently across AKI syndromes. Patients with sepsis-associated AKI may recover kidney function early but still have reduced kidney function on hospital discharge leading to dramatically reduced long-term survival [10]. Currently, no effective treatment is available to prevent this progression [23], and thus, models of AKI that develop into CKD in sepsis are essential to understand the underlying mechanisms and to develop therapies.

F4/80^+^ mononuclear cells (MNC), including macrophages and dendritic cells, function as phagocytes and antigen-presenting cells initiating immune responses. Depending on the tissue environment, these cells are highly plastic, exhibiting a broad spectrum of phenotypes, the most studied of which are the two polarized extremes, classically activated macrophages (M1) and alternatively activated macrophages (M2) [24]. The phenotypes of the infiltrated MNC contribute substantially to local immune regulation in either inflammatory or anti-inflammatory processes, closely associated with renal injury and repair [25]. Increased F4/80^+^ cells exhibiting a dominant M1-phenotype contribute to inflammation and leukocyte recruitment in the acute phases of kidney injury. By contrast, F4/80^+^ cells exhibiting an M2 phenotype play critical roles in healing and tissue regeneration during the recovery phase [26]. Our results show that, when UPHD186 is given to septic animals prior to the resolution of inflammation, decreased numbers of F4/80^+^ cells were present in the kidneys while percentages of pro-inflammatory M1 cells increased. By contrast, delaying treatment until inflammation had resolved (at least by IL-6) had no effect on the total renal F4/80^+^ cell numbers with decreased iNOS but increased MR expression (Fig. 3a–d), thus shifting the fraction of pro-inflammatory F4/80^+^iNOS^+^ downward and favoring an M2 phenotype. Our findings are in line with previous reports, whereby epigenetic regulation by HDIs could modulate numbers of mature MNC as well as their phenotype [27–29]. The novelty of this report is that MNC could be driven to transform to pro- or anti-inflammatory phenotypes, depending on the timing of HDI administration.

As readouts for S-AKI day-14 outcome, we found increased renal expression of markers which mediate inflammation (ICAM-1) injury (KIM-1) and collagen synthesis (collagen I) (Figs. 1a–e and 4d–e). Importantly, these signals were present in the kidneys following CLP even though serum creatinine was only briefly increased, and systemic inflammation marker IL-6 and renal expression of NGAL had returned to normal by 72 h. Furthermore, these changes were concurrent with maladaptive repair signs of histology impairment and fibrosis (Fig. 4d–f). Increased renal expression of both ICAM-1 and KIM-1 by day 14 represents ongoing injury in the kidney. With the renal interstitium considered as a distinct compartment [30], systemic inflammatory activation may not necessarily reflect the status in the kidney and vice versa. ICAM-1 promotes trafficking of inflammatory cells [31] and is considered a major mediator of tissue injury. In a chronic phase following AKI, epithelial cells that express KIM-1 are found in regions of unresolved injury and thus may demarcate areas of “unresolved injury/repair” in the epithelium [32]. Early treatment with UPHD186 showed wide-spread inhibition on inflammation marker mRNA expression (Fig. 3e). However, these effects unexpectedly worsened the S-AKI (histologic damage and fibrosis). This may have occurred through prematurely downregulating host defenses through inhibiting inflammatory reaction in response to pathogens [33–35], or more likely, by forcing those cells to cell cycle marked by increased *cdkn1a* (Fig. 4c), who were still coping with inflammation and not ready for regeneration. On the other hand, the beneficial effect of delayed treatment could either come from direct inhibition of collagen synthesis, fibroblast proliferation or activation [36–39], or indirect effects through modulating MNC phenotypes. These results illustrate the remarkable complexity of S-AKI with effects of treatment dependent on the pathological status at the time of intervention.

We also found IGFBP7 protein, a marker of acute kidney epithelial cell stress, was secreted into the tubular lumen in response to sepsis. The presence of secreted IGFBP7 is thought to be a manifestation of the tubular cell “secretory phenotype” that is indicative of a cell cycle arrest event upon injury [40, 41]. Consistently, clinical AKI biomarker studies have found increased urinary IGFBP7, together with tissue inhibitor of metalloproteinases-2, associated with a higher incidence of AKI compared to controls [42–44]. Furthermore, these markers have been shown to predict long-term adverse outcomes (death or dialysis) when followed by clinical evidence of AKI [45, 46]. Thus, persistent elevation of IGFBP7 likely indicates ongoing stress and may lead to maladaptive repair, given that we have previously shown upregulation of IGFBP7 in zebrafish [47] and in isolated human kidney cells [48] in response to various insults. A possible role for this molecule is as a mediator in the TGF-β1 signaling pathway [45], and it may play a key role in regulating cell migration and proliferation and increasing susceptibility of cells to apoptotic signals [49–51].

## Conclusion

In summary, we have developed and characterized a model of S-AKI that progresses to a CKD-like phenotype. We have then demonstrated that HDAC inhibition using UPHD186 was able to improve renal histology well after S-AKI. This effect appears to be mediated through a variety of mechanisms including the targeting of MNC phenotypes and inhibiting collagen synthesis. Interestingly, inhibition of inflammatory gene expression in the acute phase of S-AKI, as seen with early treatment, was not an effective treatment strategy. Conversely, delayed UPHD186 treatment improved survival, lessened the extent of renal tubular injury, and prevented interstitial fibrosis. The results of our study not only will deepen our understanding on the mechanisms of S-AKI that could direct future biomarker-guided treatment, but also suggest that treatments to improve S-AKI outcomes are complicated by time-dependency and this may affect treatment efficacy. Importantly, the HDI, UPHD186, a PTBA prodrug, may be a useful therapeutic agent for preventing the AKI to CKD transition.

## Methods

To understand whether sepsis-associated acute kidney injury could lead to renal maladaptive repair and possible influencing factors, we followed sepsis animals that treated with UPHD186 or vehicle, starting from 48 h or 96 h post-CLP, for renal outcome and measured marker expressions of maladaptive repair. The experimental setting is in vivo mice study.

### Animals

Balb/c mice (20–24 weeks old, male and female, Charles River Labs) were used. Mice were subjected to cecal ligation and puncture surgery (CLP) in accordance with methods reported by Rittirsch et al. [52]. Specifically, sepsis was induced by ligating 1-cm length of the cecum from the apex and two punctures with a 25-gauge needle. Animals in the sham group had only laparotomy without any cecal punctures. Post-surgery treatment included 40 mL/kg Ringer’s solution after surgery, and ceftriaxone (25 mg/kg), metronidazole (12.5 mg/kg), and buprenorphine (0.05 mg/kg), every 12 h administered intraperitoneally (i.p.) for 3 days. Septic animals were randomly divided into four groups to receive a 7-day UPHD186 (50 mg/kg/day, i.p.) or vehicle (20% cyclodextrin-2% DMSO in PBS, i.p.) treatment, starting from 48 h or 96 h post-CLP. To determine treatment efficacy, time serial blood samples (approx. 10 μL) were collected through animals’ saphenous vein on the days before/after the treatment session. All mice were followed for survival and sacrificed at the third day post-treatment or the endpoint day 14 to collect kidneys and blood samples.

### Kidney function and enzyme-linked immunosorbent assays

Plasma samples were collected via saphenous vein or cardiac puncture method under deep terminal anesthesia. Samples were subjected to colorimetric or ELISA assays to determine the levels of creatinine (Sigma-Aldrich), TNFα, and IL6 (R&D Systems) according to the manufacturer’s instructions.

### Immunoblotting analysis of kidney protein expression

The right kidney was harvested immediately after euthanasia, and a quarter of the kidney was minced and digested using the RIPA lysis buffer supplemented with 100 μg PMSF and 1 tablet of protease/phosphatase inhibitor (Thermo Fisher Scientific) per 10 ml buffer. After measuring concentrations using a modified DCTM protein assay, equal amounts of protein were loaded onto CriterionTM TGXTM precast gel, separated by electrophoresis, and then transferred to a nitrocellulose membrane (Bio-Rad Laboratories). After blocked 1 h at RT in PBST-5% non-fat milk, the membranes were incubated with primary antibodies at 4 °C overnight and then the corresponding secondary antibody at RT for 1 h. Images were captured and analyzed using the Odyssey Laser Fluorescence Detection System (LI-COR Biosciences). The band intensities were quantified by ImageStudioLiteVer 5.2 (LI-COR Biosciences). The primary antibodies were goat anti-NGAL (R&D Systems), rat anti-KIM-1 (R&D Systems), rabbit anti-BMPR1A (Santa Cruz Biotechnology), rabbit anti-αSMA (Abcam), mouse anti-glyceraldehyde 3-phosphate-dehydrogenase (Novus Biologicals), and rabbit anti-β-actin (Cell Signaling Technology).

### Quantitative analysis of gene expression

The total tissue RNA was isolated using TRIzol™ reagent (Life Technologies) and DNase kit (Invitrogen) and converted into complementary DNA with High-Capacity cDNA Reverse Transcription Kits (Applied Biosystems). Quantitative polymerase chain reaction was performed on a Mx3000PTM system (Agilent Technologies) using primers (Additional file 1: Table S1) and SYBR® Select Master Mix (2x) (Applied Biosystems). The expression of each target gene was assayed in duplicate and presented as fold change normalized by that of the endogenous control gene, *gapdh* expression.

### Immunofluorescence staining and immunohistochemistry

The left kidney was flushed with 10 mL ice-cold PBS, 10 mL 10% formalin through the left ventricle immediately after euthanasia, and the kidney was collected. For immunofluorescence staining, half of the kidney was fixed in 10% formalin for 2 h on ice, incubated in 30% (vol/vol) sucrose at 4 °C overnight, snap-frozen in OCT (Sakura FineTek), and cut into 7-μm sections. Sections were treated with heated citrate (Antigen Unmasking Solution, Vector Laboratories), blocked in 10% (vol/vol) normal goat serum (Vector Laboratories) in 0.3 M glycine-PBST for 1 h at RT, and incubated with primary antibodies at 4 °C overnight and then corresponding secondary antibodies (Jackson ImmunoResearch) in PBS containing 10% of the blocking solution for 1 h RT. Stained sections were mounted in a mounting medium with DAPI (Vectashield H-1000, Vector Laboratories), and stored at − 20 °C until observed under confocal microscopy (Fluoview 1000, Olympus). Three washes with PBST were performed between each step. The primary antibodies were goat anti-ICAM (R&D Systems), rabbit anti-Col1 (Novus Biologicals), rabbit anti-fibronectin (Abcam), rabbit anti-vimentin (Abcam), rat anti-F4/80 (Abcam), and rabbit anti-iNOS (Abcam), and phalloidin (Thermo Fisher Scientific) was used for staining of the brush boarder. For immunohistochemistry analysis, the infused kidney was fixed in 10% formalin at 4 °C overnight, embedded in paraffin, and cut into 5-μm sections. Hematoxylin and eosin and Masson’s trichrome stain were performed. Semiquantitative renal histology injury scoring was assessed [53, 54] by an investigator blinded to group assignment, and the percentage of fibrosis per area was quantified using ImageJ (https://imagej.nih.gov/ij/; National Institutes of Health).

### Statistical analysis

Graph and statistical analyses were performed using GraphPad Prism 8 (GraphPad Software). Numerical data are presented as mean ± standard deviation. Tukey’s test was used to determine the significance of differences among multiple groups and one-tailed Welch’s *t* test between two groups; survival rates were analyzed using the log-rank Mantel-Cox test. *P* < 0.05 was considered statistically significant.

## Supplementary information

**Additional file 1: Figure S1.** Inflammation and matrix remodeling markers were different in mRNA levels between sham and CLP. **Figure S2.** No significant differences were shown in circulating cytokines between two treatment groups. **Figure S3.** Cell cycle regulation, proliferation and pro-fibrosis markers were different in mRNA levels between sham and CLP. **Table S1.** Primers for quantitative polymerase chain reaction.

## Data Availability

The datasets generated and/or analyzed during the current study are not publicly available consistent with the University of Pittsburgh policy but are available from the corresponding author on reasonable request

## Abbreviations

7IGFBP7: Insulin-like growth factor-binding protein
BMPR1A: Bone morphogenetic protein receptor type 1A
CKD: Chronic kidney disease
CLP: Cecal ligation and puncture
Col-1: Collagen type I
HDIs: Histone deacetylase inhibitors
ICAM-1: Intercellular adhesion molecule-1
IL-6: Interleukin-6
iNOS: Inducible nitric oxide synthase
KIM-1: Kidney injury molecule-1
MNC: Mononuclear cells
MR: Mannose receptor
NGAL: Neutrophil gelatinase-associated lipocalin
PTBAs: Phenylthiobutanoic acides
S-AKI: Sepsis-associated with acute kidney injury
α-SMA: Alpha-smooth muscle actin
