# Supplementary material for: Time-dependent effects of histone deacetylase inhibition in sepsis-associated acute kidney injury
Source: Intensive Care Med Exp. 2020 Feb 7;8:9. doi: 10.1186/s40635-020-0297-3 (PMC7007462; doi:10.1186/s40635-020-0297-3)
Supplement: Supplementary file 1 — Additional file 1: Figure S1. Inflammation and matrix remodeling markers were different in mRNA levels between sham and CLP. Figure S2. No significant differences were shown in circulating cytokines between two treatment groups. Figure S3. Cell cycle regulation, proliferation and pro-fibrosis markers were different in mRNA levels between sham and CLP. Table S1. Primers for quantitative polymerase chain reaction. [file 40635_2020_297_MOESM1_ESM.docx]

**Supplemental data**

**Fig. S1.** Inflammation and matrix remodeling markers were different in mRNA levels between sham and CLP.

**Fig. S2.** No significant differences were shown in circulating cytokines between two treatment groups.

**Fig. S3.** Cell cycle regulation, proliferation and pro-fibrosis markers were different in mRNA levels between sham and CLP.

**Table. S1.** Primers for quantitative polymerase chain reaction.


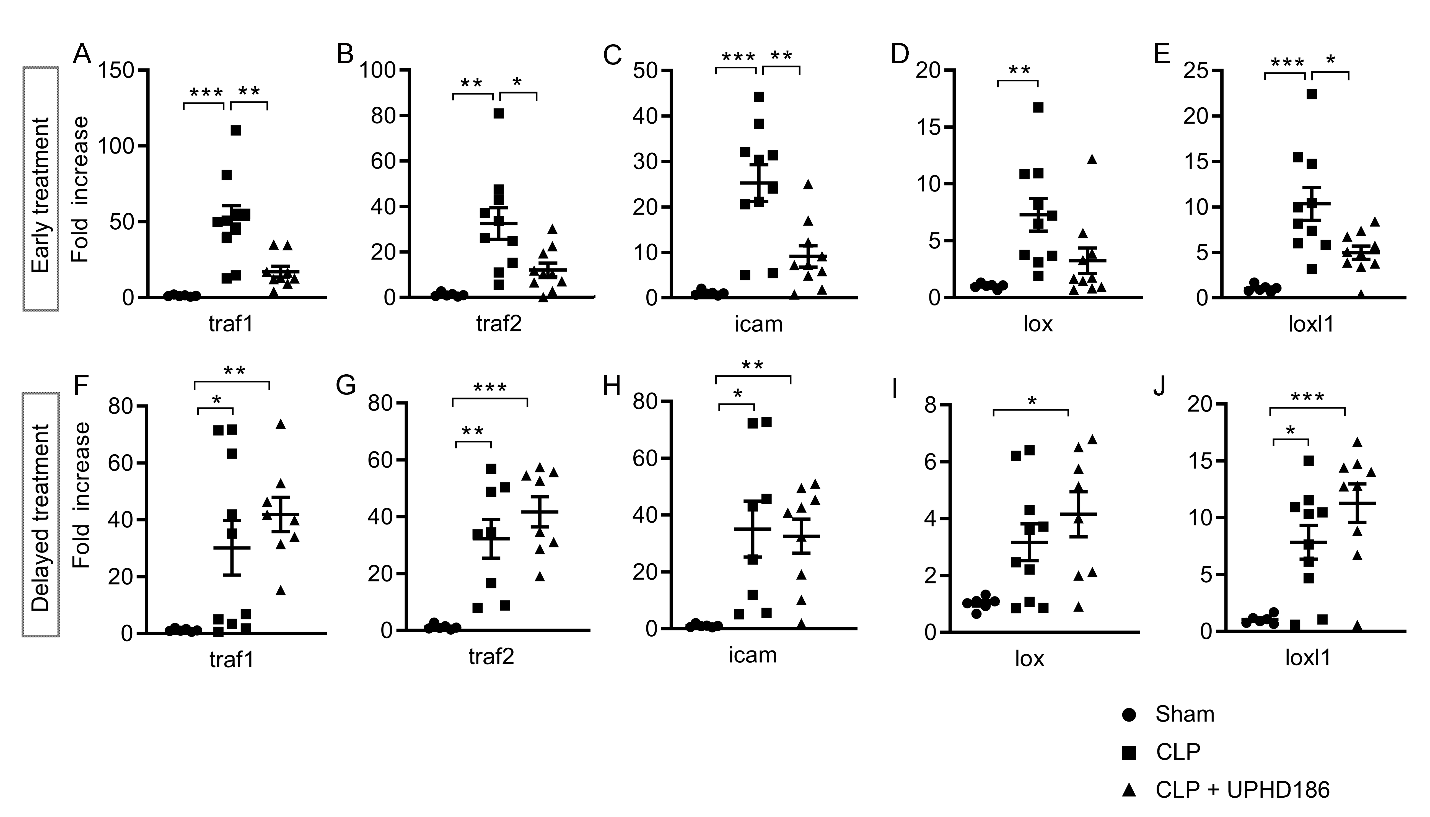


**Fig. S1.** Inflammation and matrix remodeling markers were different in mRNA levels between sham and CLP. Markers with statistically significant differences between sham and CLP + vehicle at treatment-day-3 are shown. Presented are dot plot figures with group mean ± SD of key mediators involved in inflammation (*traf1, traf2, icam*) and matrix remodeling (*lox, loxl1*). N = 6 ~ 8 for each group. **P <* 0.05, ***P <* 0.01, ****P <* 0.001. Abbreviations: *icam*, intercellular adhesion molecule; *lox*, lysyl oxidase; *loxl1*, lysyl oxidase homolog 1; *traf,* tumor necrosis factor receptor associated factor.


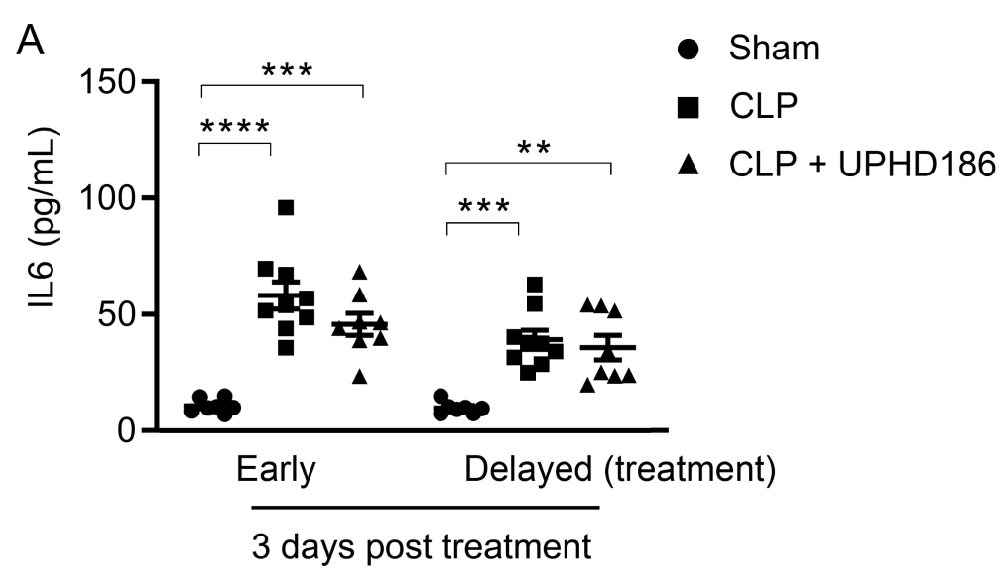


**Fig. S2.** No significant differences were seen in circulating cytokines between two treatment groups. A. dot plots of interleukin (IL) 6. N = 7 ~ 9 each group. ***P <* 0.01, ****P <* 0.001, *****P <* 0.0001. Abbreviation: Trt, UPHD186 treatment.


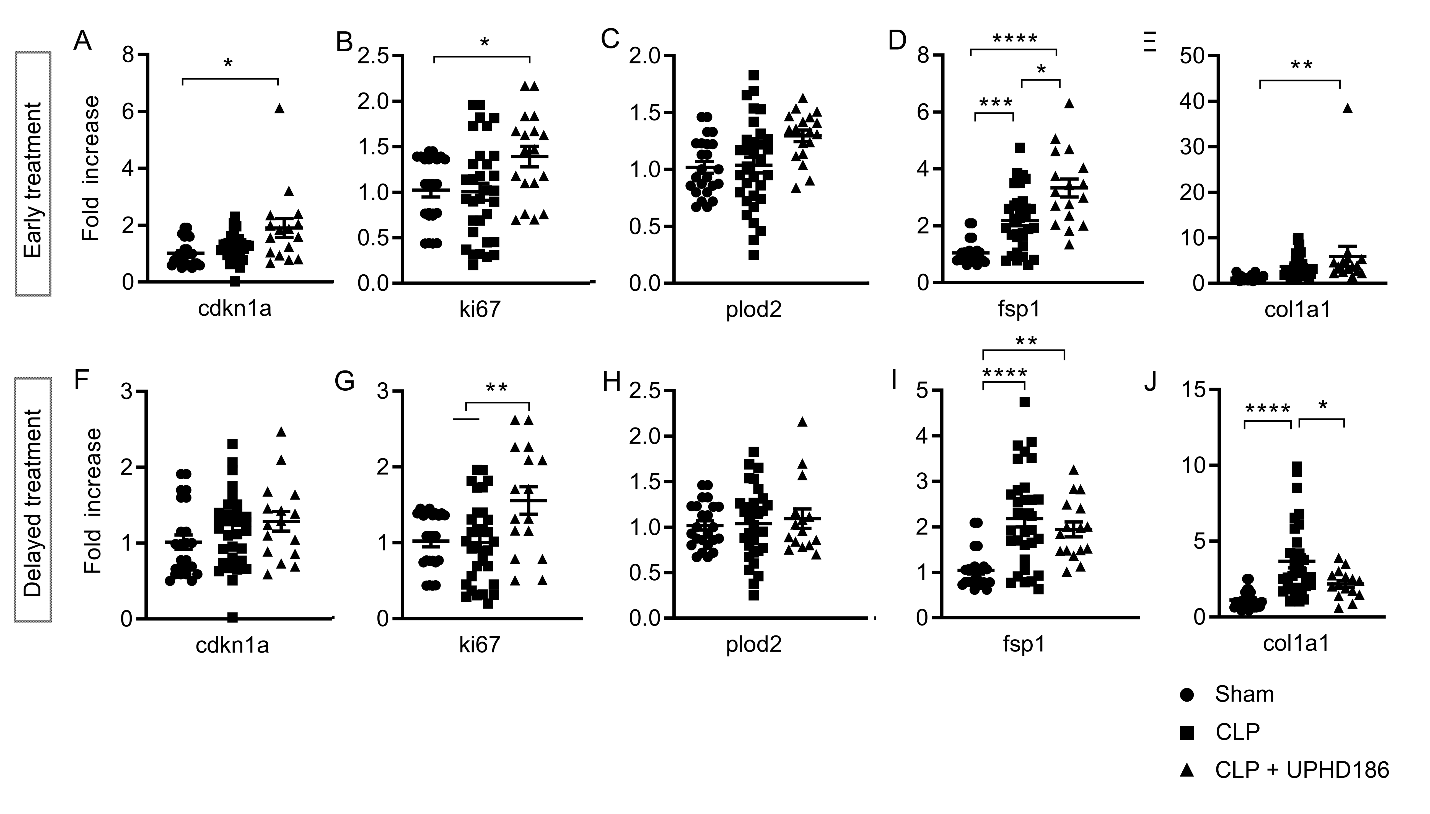


**Fig. S3.** Cell cycle regulation, proliferation and pro-fibrosis markers were different in mRNA levels between sham and CLP. Significant markers that had statistically significant differences between sham and CLP + vehicle at CLP-day-14 are shown. Presented are dot plot figures with group mean ± SD of key mediators involved in cell cycle arrest (*cdkn1a*), proliferation (*ki67*), and fibrosis (*col1a1, plod2, fsp1*). N = 9~12 for each group. *P < 0.05, **P < 0.01, ***P < 0.001, ****P < 0.0001. Abbreviations: *cdkn1a*, cyclin-dependent kinase inhibitor 1a; *col1a1*, collagen type I alpha 1 chain; *fsp1*, fibroblast-specific protein 1; *ki67*, Ki-67; *plod2*, procollagen-lysine, 2-oxoglutarate 5-dioxygenase 2.

**Table. S1.** Primers for quantitative polymerase chain reaction

| ***Gene*** | **Primer (5' to 3')** | |
| --- | --- | --- |
|  | Forward | Reverse |
| ***ccl20*** | GCCTCTCGTACATACAGACGC | CCAGTTCTGCTTTGGATCAGC |
| ***cdkn1a*** | TAGGACTCAACCGTAATATCCCGAC | AAGAGCAGCAGATCACCAGATTAAC |
| ***col1a1*** | GGTATGCTTGATCTGTATCTGCCAC | CCTCGACTCCTACATCTTCTGAGTT |
| ***fsp1*** | ACTTGGACAGCAACAGGGACA | GGGCTCCTTATCTGGGCAGC |
| ***gapdh*** | GTCAAGCTCATTTCCTGGTATGACAA | GGATAGGGCCTCTCTTGCTAGT |
| ***icam*** | GTGATGCTCAGGTATCCATCCA | CACAGTTCTCAAAGCACAGCG |
| ***ki67*** | CTGGTTGTTACTGAAGAGCCCATAC | CTTAACTGTCCTTGGTTGGTTCCTC |
| ***kim1*** | AAACCAGAGATTCCCACACG | GTCGTGGGTCTTCCTGTAGC |
| ***lox*** | CCACAGCATGGACGAATTCA | AGCTTGCTTTGTGGCCTTCA |
| ***loxl2*** | GATCTTCAGCCCCGATGGA | CAAGGGTTGCTCTGGCTTGT |
| ***nfkb*** | GGAGGCATGTTCGGTAGTGG | CCCTGCGTTGGATTTCGTG |
| ***plod2*** | GATGTTCGTTTCTGGTGGAAATTGG | TAAAGCAGAAAGACATGAGCTTCCC |
| ***traft1*** | CACTGCCAAGTATGGTTACAAGT | GGTTGTTCTGGTCAAGTAGCAT |
| ***traft2*** | TTCGGCCTTTCCAGATAACGC | ACTCCGTCAGCAGGAATGGGC |
